# Editorial: Promoting health equity via health systems transformation

**DOI:** 10.3389/fpubh.2023.1253316

**Published:** 2023-08-17

**Authors:** Weiyan Jian, Lanyue Zhang, Yan Li, Li Yang

**Affiliations:** ^1^Department of Health Policy and Management, School of Public Health, Peking University, Beijing, China; ^2^School of Public Health, Shanghai Jiao Tong University School of Medicine, Shanghai, China; ^3^Department of Population Health Science and Policy, Icahn School of Medicine at Mount Sinai, New York, NY, United States

**Keywords:** healthcare, equity, health system, health reform, health policy

Health inequity is a crucial measure of health system performance and a significant reflection of social development progress. Many countries worldwide have dedicated extensive efforts to diminish health disparities among diverse population groups (1–4). However, disparities persist due to multifaceted factors and intricate mechanisms influencing health equity. Even in developed countries, health inequity remains prevalent, and low- and middle-income countries (LMICs) face even greater challenges that necessitate sustained endeavors (5–9). The COVID-19 pandemic has exposed numerous inequalities in responses across countries and regions, emphasizing how the factors contributing to health inequity further exacerbate health risks for vulnerable groups during crises (10, 11). Despite considerable achievements in health equity over the past decade, the World Health Organization continues to emphasize the importance of “leaving no one behind,” reminding us not to underestimate the ongoing importance of this pursuit (12, 13).

This Research Topic seeks to facilitate knowledge exchange on global experiences in enhancing health service and outcome equity. It aims to provide valuable references for decision-making, improve health system performance, and enhance health service utilization for vulnerable populations worldwide. The Research Topic comprises five original articles that shed light on the convergence of wide-ranging challenges, national-level transformations, and localized strategies. By doing so, it showcases innovative strategies and potential solutions that foster health equity through systemic transformation.

Starting at the global level, the study by Cao et al. grappled with the fallout from the COVID-19 pandemic. They presented an international perspective on the pandemic's impact on health equity and offered reflections on “when and how to adjust the anti-COVID policies” across different countries. In order to achieve health equity by effective preventive policies during and beyond the COVID-19 pandemic, the authors proposed that governments should aim to keep pandemic excess mortality (PEM) within 10% when changing control strategies. Meanwhile, this study also showed that in order to reduce fluctuations in excess mortality caused by changes in policies, effective vaccination programs for vulnerable populations must be emphasized.

The study by Li et al. moved to a national perspective in an effort to enhance equitable access to medical services among vulnerable groups in rural areas. Their study confirmed that the implementation of Urban and Rural Residents Basic Medical Insurance (URRBMI) increased the fund pool and payout ratio of medical insurance for rural residents, which indeed narrowed the differences in medical service utilization between rural and urban residents without significantly increasing the hospital medical burden of rural residents. The study explored how enhancing healthcare systems through the integration and unification of health insurance programs covering different groups of people could provide a reference for other LMICs striving for health equity.

Taking a more microscopic approach, Thorndike et al. examined feasible measures to promote health equity from the perspectives of stakeholders, such as healthcare providers and service purchasers. By conducting semi-structured interviews on the “Leading Healthcare, Payment, and System Transformation” program, a roadmap to advance health equity was ultimately proposed. This roadmap included establishing a culture of health equity, identifying key areas of health equity, implementing nursing transformations and payment reforms, and constructing sustainable health equity planning. Under each track, the authors proposed several specific strategies, such as identifying key areas of health equity, using data to focus on health equity. LMICs may refer to this roadmap to build and adjust action strategies according to their actual conditions.

Bai et al. focused on another important aspect of health equity—the fair allocation of healthcare human resources. This research first confirmed that the regional inequity of China's health human resource allocation has been increasing and should attract more attention. At the same time, it found that the allocation of health human resources might be influenced by supportive resources, healthcare needs, and socio-economic and cultural factors in neighboring regions. The findings highlighted the importance of considering local dynamics in the planning and implementation of equitable health resource allocation. It further emphasized the need for systemic equity, now on a localized scale.

Finally, we narrowed our focus to a specific population group with Liang et al. exploration of the impact of nutrition during adolescence. This research showed that a nutritional improvement program for the compulsory education of rural students reduced the incidence of malnutrition and growth retardation in teenagers by 9% in the short term, preventively improving regional health disparities. This research supplemented the empirical results from China based on studies in countries such as India and the United States, emphasizing how specific interventions could address health disparities. This study was a testament to the significant impact local interventions can have on broader health equity goals.

In conclusion, these diverse studies underscore the importance of adopting a multi-tiered approach to foster health equity, encompassing global, national, and local strategies. Collectively, they illuminate a clear pathway toward transformative changes in health systems, recognizing the intricate interconnectedness of global crises, national policies, and local interventions.

## Author contributions

WJ: Conceptualization, Writing—original draft. LZ: Writing—original draft. YL: Supervision, Writing—review and editing. LY: Supervision, Writing—review and editing.
